# Online peer-led intervention to improve adolescent wellbeing during the COVID-19 pandemic: a randomised controlled trial

**DOI:** 10.1186/s13034-024-00723-1

**Published:** 2024-03-18

**Authors:** Gabriela Pavarini, Tessa Reardon, Geoffrey Mawdsley, Ilina Singh

**Affiliations:** 1 Ethox Centre, Oxford Population Health, University of Oxford, Old Road Campus, Oxford, OX37LF UK; 2 Department of Experimental Psychology, University of Oxford, Oxford, UK; 3 Department of Psychiatry, University of Oxford, Oxford, UK; 4 Wellcome Centre for Ethics and Humanities, University of Oxford, Oxford, UK

**Keywords:** COVID-19, Peer-led intervention, Peer support, Mental health, Wellbeing, Coping, Empowerment

## Abstract

**Background:**

The COVID-19 pandemic and associated lockdown measures have posed a major risk to young people’s wellbeing, which might be ameliorated by peer-led programmes. Using a randomised controlled trial (ISRCTN registry, number ISRCTN77941736 https://doi.org/10.1186/ISRCTN77941736), we tested the short-term efficacy of an online peer-led intervention designed to equip young people with skills to support their mental health and wellbeing during the COVID-19 pandemic.

**Methods:**

Through schools and social media ads, we recruited one hundred young people (aged 16–18) in the UK, focusing on areas with the highest incidence of COVID cases. In December 2020, participants were randomly allocated (1:1) to immediate 5 day Coping during COVID course (n = 49) or a wait-list (n = 51) through a survey software automated randomisation tool. Our primary outcome was self-reported mental wellbeing, and secondary outcomes included self-reported social connectedness, coping skills, sense of purpose, self-esteem, and self-compassion. We also collected qualitative reports of participants’ perceived impact of the course and intentions to use what they have learnt from the course in their life moving forward. Assessments were completed at baseline, 1 week post randomisation (primary endpoint), and 2-weeks post-randomisation.

**Results:**

Young people allocated to the peer-led intervention reported significantly greater wellbeing, social connectedness, coping skills, sense of purpose, self-esteem, and self-compassion 1 week and 2 weeks post-randomisation (medium-large effect sizes). Specific benefits to mental health, sense of purpose and connectedness were also emphasised in qualitative reports.

**Conclusions:**

An online, peer-led intervention targeting youth wellbeing during the context of the COVID-19 pandemic brought benefits across a range of outcomes, suggesting that structured programmes that incorporate peer-to-peer support can be a valuable approach to promote young people’s wellbeing and foster psychological resources during a health crisis.

**Supplementary Information:**

The online version contains supplementary material available at 10.1186/s13034-024-00723-1.

## Key points

- Although the potential benefits of peer-led interventions are widely postulated, the evidence base on their effectiveness in improving peer recipients’ wellbeing remains limited.
- Responding to young people’s expressed priorities during COVID-19, this study investigated the impact of participating in an online peer-led intervention designed to support young people’s wellbeing during the pandemic.
- Peer-led intervention (vs. waitlist control) increased adolescents’ mental wellbeing, social connectedness, coping skills, sense of purpose, self-esteem, and self-compassion
- Peer-led intervention seems to be a valuable tool to support young people’s mental wellbeing during a health crisis.

**Supplementary Information:**

The online version contains supplementary material available at 10.1186/s13034-024-00723-1.

## Introduction

The COVID-19 pandemic and associated mitigation efforts have posed multiple challenges to young people’s mental health and wellbeing, including feelings of anxiety, loneliness, and lack of purpose [1–3]. Despite longstanding sociological accounts of childhood that theorise young people as citizens and social actors in their own right [4], young people have been largely sidelined in the COVID-19 crisis response. In the UK, the voices of young people have been noticeably absent from key decisions, such as school closures and changes in educational assessment procedures, giving rise to feelings of exclusion and lack of control [5, 6]. In the context of mental health, while numerous research projects tracked outcomes for young people [7], more investment and effort could be made to support adolescents to take an active role in both the design and delivery of mental health and wellbeing interventions during this time.

Adolescent involvement in the delivery of interventions targeting young people’s mental health and wellbeing is a widely used approach [8]. Peer-led interventions typically involve selecting and training a group of adolescents to provide emotional support to similar-aged youth and/or teach them key skills to manage their mental health. This is implemented through dyadic or group-based interactions, often with guidance and support from adults. While peer-led interventions are often used as a tool to support adolescents’ mental health and wellbeing in schools [9, 10], online approaches have attracted growing attention [11]. Researchers have specifically highlighted their potential benefits during the context of the COVID-19 pandemic, given social distancing requirements separating young people from their typical support networks [12–15].

Peer-led interventions are a promising approach for multiple reasons. During adolescence, emotional investment is reoriented from parents to peers, increasing self-disclosure within peer networks [16]. Young people tend to turn to peers for support and comfort [17] and positive peer relationships are associated with improved mental health and wellbeing [18]. Within peer-led interventions, peer leaders are perceived by supported peers as assuming a dual role of equal and mentor—someone who is relatable and provides companionship while also acting as a role model [19]. This provides a promising context to learn socioemotional skills, especially given young people’s high sensitivity to peers’ recognition and influence [20, 21].

Despite these benefits, current evidence to support the positive outcomes of peer-led interventions for young people’s mental health and wellbeing is limited and inconclusive. In a recent review of peer-led school interventions, only two out of five identified studies documented benefits for supported young people’s self-confidence and quality of life, with one suggesting a negative impact on general mental health [22]. Similarly, in a systematic review of digital interventions, only two out of five studies, which evaluated structured interventions targeting anxiety and tobacco use, demonstrated positive mental health outcomes. The remaining studies, mostly involving unmoderated online boards, yielded null results [23]. Training and support from adult stakeholders for peer leaders may play a crucial role in achieving positive outcomes for the recipients of peer-delivered programs. Importantly, neither of the reviews reported that the training provided to peer leaders was evidence-based. Furthermore, the type and extent of training and support for peer leaders varied considerably across the studies.

Considerable heterogeneity across existing studies both in relation to study design (e.g., target population, outcomes assessed) and intervention characteristics (e.g., intervention content, delivery format) makes it difficult to draw firm conclusions about the efficacy of peer-led interventions, even in the short term. Indeed, both existing reviews pointed to the need for randomised controlled studies to test the impact of peer-led interventions on supported peers, since much of the evidence base consists of quasi-experiments or pre-post designs. Moreover, to our knowledge no studies have designed or evaluated peer-led interventions targeting young people’s mental health and wellbeing either in the context of the COVID-19 pandemic or other health crises.

Another key shortcoming of the current literature is the lack of youth involvement in the *design* of peer-led interventions and intervention evaluation studies. Many existing studies only involve adolescents at the implementation stage (see [23]), yet several researchers highlight the importance of building ownership and engaging young people in the development and evaluation of new interventions [24–26]. Involving young people as active stakeholders in designing interventions can help ensure youth-friendly programmes that address adolescents’ specific mental health needs [27, 28]. Active youth involvement also supports young people’s right to citizenship and participation, particularly during a time of deep uncertainties such as the COVID-19 pandemic [6, 29].

In the present study, our research team partnered with peer support specialists from the charity Youth Era (www.youthera.org) and young people to develop a peer-led, online intervention (*Coping During COVID*) designed to target the socioemotional challenges of the COVID-19 pandemic from the perspective of UK youth. *Coping During COVID* is a group-based course, co-delivered by adolescents (peer leaders) in partnership and with support from adult peer support specialists. Peer leaders delivering the course received training in online peer support through an evidence-based programme called “Uplift”, trialled in a previous study [30]. *Coping During COVID* was designed to provide young people with the opportunity to connect and share experiences with an online peer community during the pandemic, while learning emotional coping skills.

Through a randomised controlled trial, we investigated whether *Coping during COVID* improved the wellbeing of young people aged 16–18 years living through the COVID-19 pandemic in the UK. We also investigated whether the intervention had benefits in relation to additional intervention-targets, including young people’s social connectedness to peers, emotional coping skills, sense of purpose, self-esteem and self-compassion. These outcomes were all identified as key for young people’s mental wellbeing during the COVID-19 pandemic during initial consultations with groups of UK adolescents aged 16–18 years at the start of the pandemic [30]. Given that this is the first evaluation of this intervention, and that immediate effects of peer-led interventions have not yet been established [22, 23], we investigated short-term benefits to supported peers, relative to a wait-list control. Through open questions we also investigated young people’s perceived impact of the intervention in their lives, and young people’s plans to use the skills they learnt in the future.

## Materials and methods

### Patient and Public Involvement

This project was supported by two Young People’s Advisory Groups: the NeurOX YPAG, a group of young people aged 14–18 years who support research in ethics and youth mental health [31], and the Uplift YPAG, set up specifically for this project. Uplift YPAG members were recruited from a larger group of young people aged 16–18 years trained in peer support in a previous project [30], on the basis of suitability and readiness to deliver peer support (assessed by professional judgement from Youth Era) and availability. Priority was given to trained young people from ethnic minority backgrounds and low family affluence. The YPAGs provided extensive input into the course design, including overall structure, content and delivery methods, and Uplift YPAG members additionally acted as peer leaders. Both groups provided input into trial design (e.g., recruitment strategies, data collection methods, outcome measures) and results interpretation (see Additional file 1 for further details on young people’s contributions). All YPAG members were UK residents from a range of socioeconomic and ethnic backgrounds.

### Recruitment

#### Participants and recruitment context

The study protocol was approved by the University of Oxford Interdivisional Medical Sciences Ethics Committee (R69810/RE001). Participants were recruited through social media adverts and schools across the UK between the 14th of November 2020 and 2nd of December 2020. The poster/advert invited young people to take part in a ‘Coping during COVID’ online course while contributing to research. The aim was to reach at nation-wide sample of adolescents experiencing common emotional difficulties during this period. Eligibility criteria for the study included being aged 16–18, UK resident, a sufficient level of English, consenting to random assignment to one of two iterations of the *Coping during COVID* course, access to a device with camera, sound and microphone. Those interested were directed to an “Expression of Interest” form via an online survey platform. Potentially eligible participants were invited for a call with Youth Era staff where eligibility and suitability were confirmed. Once confirmed, written informed consent and baseline measures were obtained via the Qualtrics online survey platform. Participants were incentivised to complete the surveys at three assessment points with a £20 voucher reimbursement at the end of their participation.

During the time of data collection, England was operating a three-tier system, with stricter restrictions for areas of high incidence; however, schools, shops and personal care centres were open across three tiers. Social distancing was enforced, and people were encouraged to stay in their local areas as far as possible. To reach young people for whom the intervention would be most valuable and relevant, social media adverts targeted areas classified as Tier 2 and Tier 3, as well as areas of high incidence across Northern Ireland, Wales and Scotland. Once these places were filled, we also set adverts and contacted schools in other areas in the UK to reach our recruitment target.

#### Procedure

After completion of the baseline measures, participants were automatically randomly allocated in a 1:1 ratio to intervention or wait-list control using the Qualtrics randomisation tool. We chose to use this randomisation procedure because feedback from participants in a previous trial indicated that young people wished to know their course allocation immediately, to allow them to save the dates and prepare for the intervention. The automated tool ensured that the research team could not affect randomisation. Participants randomised to the intervention arm completed the course from the 2nd-6th of December 2020 (course 1) whilst the wait-list control were offered to complete the course after the final assessment from the 16–20th of December 2020 (course 2). Both arms were assessed via Qualtrics surveys immediately post-course (approximately 1 week post-randomisation) and at a follow-up point 1 week post-course (approximately 2 weeks post-randomisation). Participants completed all the assessments independently online; surveys were distributed via email following standard wording, by a researcher blind to condition. Survey answers were identified by a randomly assigned ID and participants were made aware that neither Youth Era nor their peer leaders had access to their answers.

#### Trial registration and deviation from protocol

The trial was registered under ISRCTN77941736 (https://doi.org/10.1186/ISRCTN77941736). Because of the rapid planning and delivery of this project in the context of the COVID-19 pandemic, the trial registration was only submitted shortly before recruitment started (12-Nov-2020, with recruitment commencing 14-Nov-2020) and therefore registration was published during the recruitment period as retrospectively registered (23-Nov-2020). Participant recruitment was slower than anticipated and as a result we made two changes to the original protocol. Firstly, we changed the second follow-up from 2 weeks post-course to 1 week post-course. This allowed us to extend our recruitment period without extending the overall trial duration and ensured follow-up assessments were complete before the Christmas holiday period. Secondly, we reviewed our sample size calculation and reduced the target sample size from 120 to 100 participants (see *Statistical analysis*).

#### Coping during COVID intervention and peer leader training

*Coping during COVID* was co-delivered to participants via Zoom by a selected group of young people from the Uplift YPAG group (peer leaders) and a team of specialist Youth Era staff, over 5 consecutive days (2 h/weekday; 4 h/weekend day). The course was made up of educational lectures delivered by Youth Era staff to the whole group, and small group activities and supportive and sharing sessions, led by the peer leaders. Small groups consisted of 5–7 participants, with mixed gender and age, and small group activities were delivered using both Zoom and WhatsApp. Participants were unacquainted to their peer leaders prior to the course.

The course focused on self-care, coping with emotions, identifying strengths, building resilience, improving coping skills, developing purpose, and forming community. Activities included, for instance, generating coping strategies for a variety of scenarios; identifying and challenging self-limiting beliefs; sharing letters of appreciation among group members; and drafting a self-care plan. More details of the course content are provided in Additional file 2. Prioritisation of course content was influenced by data from the literature and YPAG input on what young people were struggling with during that current period of the COVID-19 pandemic. Even though the socioemotional skills targeted (e.g., coping) were broadly relevant, COVID-specific cases and examples were provided where possible. For example, icebreaker questions included “What have you learned about yourself during COVID?”.

Of the group of Uplift YPAG members who co-designed the course (21 members), 9 co-delivered the course for the intervention arm based on their availability. Two additional youth acted as background support, providing one-to-one peer support when necessary and assisting with large group activities. The remaining members of the Uplift YPAG acted as peer leaders to the waitlist control arm.

Peer leaders had all received previous training in peer support through the Uplift Peer Support Training Programme. This training course equipped adolescents with the skills to support the mental health and wellbeing of friends and peers during the COVID-19 pandemic. Young people who received this training reported greater ability to help others, compassion and civic engagement, compared to controls [30]. Peer leaders also received an additional 2-day training provided by Youth Era before delivering the *Coping During Covid* course in the current trial. Peer leaders’ age range matched that of adolescents who took the *Coping during COVID* intervention (i.e. trial participants): 16–18 years old.

Peer leaders received close support and mentorship from Youth Era staff throughout. This included daily meetings before and after the course to provide guidance and discuss any difficulties. Youth Era staff were also on call during the small group activities in case peer leaders had an emergency or needed support.

### Outcome measures

#### Primary and secondary outcomes

All quantitative outcomes were assessed in the intervention and control group using self-report measures at baseline, 1 week and 2 weeks post-randomisation. Our primary outcome was wellbeing at 1 week, and secondary outcomes included connectedness, perceived coping skills, sense of purpose, self-esteem and self-compassion at 1 week and 2 weeks, and wellbeing at 2 weeks.

Wellbeing was measured using the Warwick-Edinburgh Mental Wellbeing Scale (WEMWS; [32]), which includes 14 items (e.g., “I’ve been feeling relaxed”; total score: 14–70).

Connectedness was measured using the Social Connectedness Scale [33], including 20 items, adapted to refer to peer relationships (e.g., “I feel close to my peers”; total score: 20–120).

We measured perceived coping skills using items adapted from the COVID-19 Adolescent Symptom & Psychological Experience Questionnaire [34]. The questionnaire included three items about participants’ confidence in their ability to manage negative emotions arising from the COVID-19 pandemic and lockdown measures (total score: 3–15).

Sense of purpose was measured using the 12-item Claremont Purpose Scale [35], which assesses three dimensions of purpose: meaningfulness, goal orientation, and a dimension called “beyond the self” which was modified to assess participants belief in their *ability* (rather than motivation) to make a meaningful contribution to the world (total score: 12–60).

To measure self-esteem, we used Rosenberg’s 10-item Self-esteem Scale [36] (e.g., *“*On the whole, I have been satisfied with myself”; total score: 10–40).

Self-compassion was measured using the Self-Compassion Scale [37], including the 6-item Compassionate Engagement Subscale (e.g. “I have been accepting, non-critical and non-judgemental of my feelings of distress”) and the 4-item Compassionate Action Subscale (“I have directed my attention to what is likely to be helpful to me”) (total score: 10–100).

#### Qualitative outcomes

Open-ended questions immediately post-intervention (1 week post-randomisation) captured participants’ experience of the course, including perceived impact, and plans to implement course content. Participants allocated to the intervention arm were asked: i) how the course impacted their life, either positively or negatively (e.g., how they see themselves, their relationships, day-to-day life), and ii) their plans to use what they have learnt from the course in their life moving forward. Additional questions about views and experience of the course itself (e.g., suggested improvements) and responses to situations when participants felt distressed or upset were also included. Results from these additional questions fall outside the scope of this paper and will not be reported here.

### Statistical analysis

#### Sample size

Our original target sample size was 120 participants and was determined on the basis that with a retention rate of 90% this would be provide > 85% power at the 2 sided 5% significance level to detect a difference between the intervention and wait-list group on the primary outcome of 0.6 standard deviations (Cohen’s d medium effect size). Participant recruitment was slower than anticipated so during the recruitment period we took the pragmatic decision to adjust our recruitment target to 100 participants on the basis that with a 90% retention rate this sample size would still provide 80% power to detect the planned effect size (0.6). G power was used for sample size calculations.

#### Analysis

Descriptive statistics (mean, SD; n, %) were used to summarise baseline demographic characteristics for each group. We used linear mixed effects models to compare the two groups over time for each outcome variable. Each model included fixed effects of group (intervention, control), timepoint (baseline, 1 week, 2 week), group by timepoint interaction, gender, age, and corresponding baseline score. To account for the fact that participants completed measures over multiple time points, a random participant effect (random intercept) was included in each model. Primary and secondary outcomes were analysed using intention-to-treat principles, without imputation. For each outcome, we present the difference in estimated means between groups for each time point, together with 95% CI for the difference, p-value, and effect size (Cohen’s d).

### Qualitative analysis

We adopted a directed content-analysis approach for qualitative analysis of responses to open-ended questions related to impact of the course and intentions to use skills. The analysis was guided by literature on potential positive outcomes associated with peer-led interventions. New codes emerging from the data were also identified and included as appropriate. The initial coding frameworks was developed by GM based on responses related to course impact, and further iterated through meetings with GP and TR. The same scheme was then applied to intentions to use what they have learned, with additions when new content was identified. The final coding frameworks were applied to the full dataset, where we coded the presence/absence of each code for each response, regardless of response length. A second coder additionally analysed 25% of the data, reaching substantial inter-rater agreement (κ =  > 0.704) [38].

## Results

### Participants

**Fig. 1:**
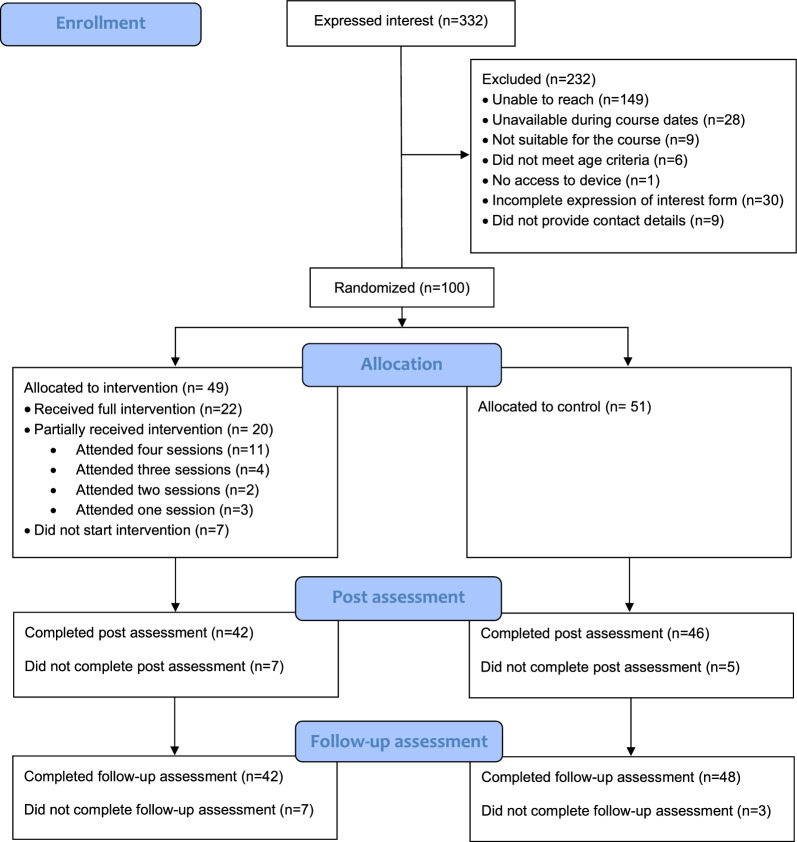
Participants’ progress through the trial

One hundred participants were randomised to the intervention group (n = 49) or waitlist (n = 51)^1^ Baseline characteristics are presented in Table 1 and retention rates are provided in Fig. 1. Most participants were aged 16 years, lived in England, identified their gender as woman/girl, and identified their ethnicity as White British or Asian. Most participants had medium family affluence as measured by the Family Affluence Scale, an adolescent-friendly assessment of socio-economic status based on material markers [39].

**Table 1:** Baseline demographic characteristics

	Intervention(n = 49)	Wait-list control (n = 51)
Age		
16 years, n (%)	31 (63.3)	37 (72.5)
17 years, n (%)	17 (34.7)	13 (25.5)
18 years, n (%)	1 (2.0)	1 (2.0)
Gender (n, %)		
Woman/girl	42 (85.7)	41 (80.4)
Man/boy	5 (10.2)	9 (17.6)
Non-binary	2 (4.1)	1 (2.0)
Family affluence scale (4 items)		
Mean (SD)	5.59 (1.81)	5.41 (1.53)
Low affluence (score 0–3), n (%)	7 (14.3)	6 (11.8)
Medium affluence, (score 4–6) n (%)	25 (51.0)	37 (72.5)
High affluence, (score 7–9) n (%)	17 (34.7)	8 (15.7)
Ethnicity (n, %)		
White British	20 (40.8)	10 (19.6)
White Irish/White other	4 (8.2)	3 (5.9)
Black African/Black Caribbean/other Black background	7 (14.3)	9 (17.6)
Mixed background	4 (8.2)	2 (3.4)
Indian/Pakistani/Bangladeshi/Chinese/Other Asian background	14 (28.6)	23 (45.1)
Other ethnic group	0 (0)	4 (7.8)
Location		
England	44 (89.8)	45 (88.2)
Scotland	2 (4.1)	2 (3.9)
Wales	1 (2.0)	3 (5.9)
Northern Ireland	2 (4.1)	1 (2.0)

### Quantitative results

Baseline, 1 week and 2 week post randomisation outcomes for the intervention and control groups are detailed in Table 2, and mixed effects models showed a significant main effect for group, timepoint, and group by timepoint interaction for each primary and secondary outcome (See Additional file 1). Self-reported wellbeing, connectedness to peers, perceived coping skills, sense of purpose, self-esteem, and self-compassion were each significantly higher among the intervention group than the control group 1 week and 2 weeks post-randomisation (Table 3). Effect sizes were medium to large across outcomes (d = 0.55 to d = 1.48), with the effects larger at 1 week than 2 weeks post-randomisation for each outcome.

**Table 2:** Baseline, 1 week and 2 weeks post-randomisation outcomes for intervention and wait-list control groups

	Course (N = 49)	Wait-list (N = 51)
Wellbeing		
Baseline, mean (SD), *n*	42.57 (7.34), *n* = 49	44.45 (7.43), *n* = 51
Post, mean (SD), *n*	50.62 (8.89), *n* = 42	42.98 (9.14), *n* = 46
Follow-up, mean (SD), *n*	48.05 (9.96), *n* = 42	44.27 (9.61), *n* = 48
Social connectedness		
Baseline, mean (SD), *n*	74.69 (16.93), *n* = 49	72.71 (17.86), *n* = 51
Post, mean (SD), *n*	92.29 (16.59), *n* = 42	74.07 (19.79), *n* = 46
Follow-up, mean (SD), *n*	87.02 (16.75), *n* = 42	78.45 (18.00), *n* = 47
Perceived coping skills		
Baseline, mean (SD), *n*	8.55 (2.18), *n* = 49	8.84 (2.48), *n* = 51
Post, mean (SD), *n*	11.24 (2.21), *n* = 42	9.74 (5.58), *n* = 46
Follow-up, mean (SD), *n*	11.00 (2.75), *n* = 42	9.96 (2.56), *n* = 47
Self-compassion (total)		
Baseline, mean (SD), *n*	59.15 (12.88), *n* = 49	59.37 (12.90), *n* = 51
Post, mean (SD), *n*	67.05 (15.35), *n* = 42	59.37 (11.75), *n* = 46
Follow-up, mean (SD), *n*	64.62 (17.07), *n* = 42	57.63 (15.04), *n* = 47
Self-esteem		
Baseline, mean (SD), *n*	24.65 (4.52), *n* = 49	25.20 (5.37), *n* = 51
Post, mean (SD), *n*	28.64 (5.13), *n* = 42	25.13 (6.14), *n* = 46
Follow-up, mean (SD), *n*	27.95 (5.86), *n* = 42	26.28 (6.45), *n* = 47
Sense of purpose (total)		
Baseline, mean (SD), *n*	37.78 (10.05), *n* = 49	38.12 (9.51), *n* = 51
Post, mean (SD), *n*	45.07 (10.04), *n* = 42	35.26 (9.10), *n* = 46
Follow-up, mean (SD), *n*	43.83 (11.28), *n* = 40	36.55 (10.84), *n* = 47

**Table 3:** Difference in estimated means* (intervention-control) and 95% CI for primary and secondary outcomes

	Wellbeing	Social connectedness	Perceived coping	Self-esteem	Sense of purpose (total)	Self-compassion (total)
1 week post-randomisation mean difference	8.87 (6.38–11.35), p < 0.001, d = 1.38	16.31 (12.07–20.55), p < 0.001, d = 1.48	1.76 (0.94–2.57), p < 0.001, d = 0.83	4.07 (2.74–5.40), p < 0.001, d = 1.18	9.53 (7.09–11.96), p < 0.001, d = 1.50	7.61 (3.31–11.91), p = 0.001, d = 0.67
2 week post-randomisation mean difference	5.17 (2.70–7.63), p < 0.001, d = 0.79	6.53 (2.32–10.75), p = 0.003, d = 0.59	1.21 (0.40–2.02), p = 0.004, d = 0.57	2.28 (0.96–3.61), p = 0.001, d = 0.66	6.73 (4.28–9.19), p < 0.001, d = 1.05	6.27 (1.99–10.55), p = 0.004, d = 0.55

^a^Adjusted for gender, age, corresponding baseline score

#### Impact and intentions to use skills

**Fig. 2:**
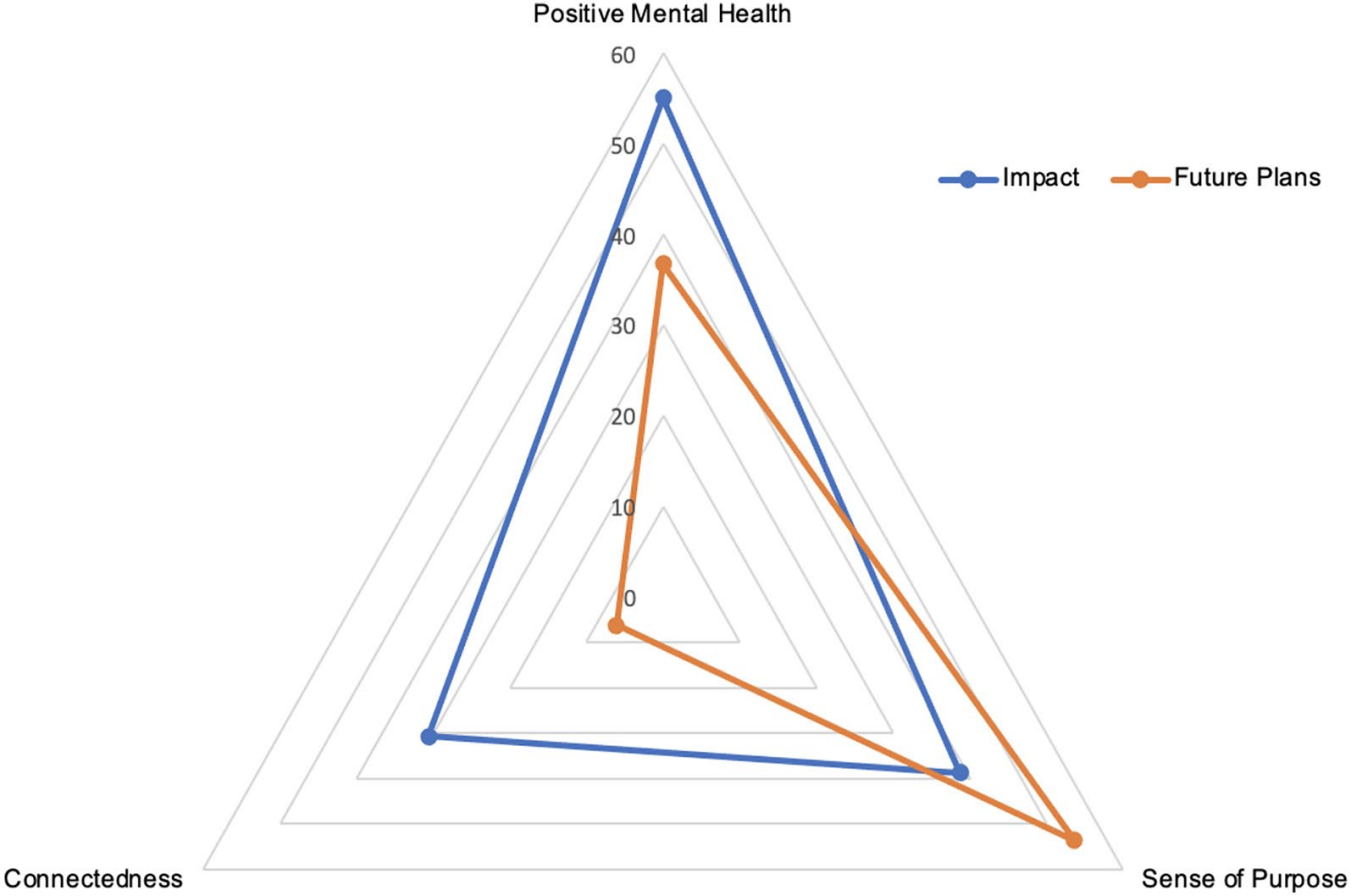
Percentage of participants who mentioned each type of content: positive mental health, peer connectedness and sense of purpose

Three themes were identified from intervention participants’ responses across the open-ended question regarding the impact of the programme: positive mental health, sense of purpose, and peer connectedness (see Fig. 2 for proportion of participants who mentioned each type of content).

Over half of the participants made references to aspects of positive mental health, including having learnt useful skills and strategies to improve one’s mental health (e.g., coping skills, self-care) as well as seeing direct improvements in their self-esteem, confidence, hopefulness and general wellbeing.

*“I now know how to self-care. I used the wheel [a visual self-care rating scale] that we were taught to see how my week has been and what I need to improve for this week coming” (Zoe, 17 years old; names are pseudonyms)*

*“It has definitely impacted my life in the way that I see myself and hold myself, I've become more confident and definitely now come across as a more confident person” (Jessica, 17)*

About two fifths of the participants mentioned that the course impacted their sense of purpose, including their perceived capabilities to make a difference and offer support to others.

*“I see myself as a valuable part of society and that my opinions matter” (Holly, 17) *

*“The training has taught me how to make other people feel more appreciated and listen to what they have to say better” (Aysha, 16)*

Finally, about a third of responses reflected on the impact of the course on peer connectedness, including the development of new connections and friendships made on the course as well as impacting existing friendships.

*“The relationships I have made through the course have also positively impacted me as I now have more friends” (Poppy, 16)*

*“I’ve reconnected with a lot of friends who I lost contact with over the lockdown periods” (Maria, 17)*

When asked to reflect on plans to use what they learnt, the same three themes of positive mental health, sense of purpose, and peer connectedness were identified (see Fig. 2 for percentages), in addition to a fourth, less common theme surrounding professional and academic aspirations e.g. “I’m willing to apply what I learned into my future career” (Rashida, 17). While references to aspects of positive mental health and connectedness were not as frequently mentioned in this section as under perceived impact, particularly prominent were references to a regained sense of purpose—most participants mentioned a desire to implement what they had learnt to help friends and family, and to give back to the community at large.

*“I’m going to use when talking to my other friends when they are having any problems (...) show them that they are not alone and that it's normal to feel down at times” (Sienna, 16).*

*“I would like to go on to help people that have been struggling with motivation and feelings of worthlessness. I am considering finding further support training courses” (Sophie, 18)*

## Discussion

Our randomised controlled trial tested the efficacy of a peer-led intervention to support young people’s wellbeing during the COVID-19 pandemic, delivered online and in groups over a period of 5 days. The intervention increased young people’s wellbeing, as well as their social connectedness, emotional coping skills, sense of purpose, self-esteem and self-compassion, compared to waitlist controls. Consistent with the quantitative results, participants in the intervention group described that they learnt helpful strategies to promote their mental health and wellbeing, gained a sense of connectedness to peers, and improved their sense of purpose. Participants expressed intentions to apply what they had learnt in their lives moving forward, not only to promote their own wellbeing but also to help their friends, peers, and community at large. These results complement our previous trial [30], which demonstrated the efficacy of the Uplift Peer Support Training Programme on young people’s perceived ability to help others and self-reported compassion. We now document benefits to adolescents *receiving* support from these trained adolescents through a structured peer-led programme.

Online interventions that provide an opportunity for young people to learn socioemotional skills are critical in the pandemic context, given the high incidence of mental distress, including anxiety, grief and uncertainty [1–3]. The chance to connect with a group of peers might also help alleviate negative psychological effects of COVID-19 mitigation measures, given the potential harmful effects of physical distancing and restricted social contact for adolescent mental health [40].

The benefits of ‘Coping during COVID’ may extend beyond the pandemic context, as it addresses prevalent challenges. Indeed, our findings are consistent with positive outcomes reported in previous evaluations of online peer-led interventions for adolescents [23]. Notably, previous interventions showing positive results incorporated structured peer-to-peer support sessions and training and mentorship from experts for peer leaders, distinguishing them from spontaneous support in online forums. More specifically, two previous trials indicated that one-to-one online interventions delivered by trained peer counsellors were effective in reducing symptoms of anxiety [41] and supporting smoking cessation [42]. Our findings add to this literature, demonstrating that a short, structured, group online intervention can also produce benefits for adolescent wellbeing and related outcomes, including social connectedness and sense of purpose.

Critically, the *Coping During COVID* intervention inspired adolescents to use the skills they learned to help their peers and community in the future. This might have been due to the fact that the intervention directly invited participants to reflect on their sense of purpose, but it is also possible that the peer leaders provided a positive, prosocial role-model for those receiving the intervention. This is in line with previous research suggesting that during middle-adolescence young people are susceptible to prosocial influence from peers [20, 21]. To facilitate a potential ripple effect in peer communities, future interventions could integrate peer leader training at the end of the intervention, to prepare adolescents to “pass on” what they learnt. Such “train-the-trainer” models have been used effectively to support sustainability of other types of intervention, for instance in the field of sexual and reproductive health [43].

### Youth-adult partnerships and future directions

Our study was driven by priorities voiced by young people at the start of the pandemic [30], and involved groups of adolescents in the design of the intervention and throughout the research process. Such extensive youth involvement was key to ensure the programme and research met young people’s specific needs and felt meaningful to them. It is possible that close and continued youth involvement is an important factor in determining whether peer-led interventions are effective, a possibility that should be explored in future research to help reconcile mixed findings.

Future research should also explore the impact of adult involvement in training and delivering peer-led programmes. We believe that the adult support peer leaders received during the course was critical for the safe and effective delivery of the intervention during an emotionally challenging time. Subsequent studies should investigate mediators of peer-led intervention effects across various outcomes to identify critical intervention components and determine the optimal level of training and adult support.

As our study focused on adolescents within a narrow age range, with equivalent ages between peer leaders and supported peers, it is uncertain whether comparable outcomes would emerge in different age groups or mixed-age settings. Additionally, we recruited previously unacquainted youth across the UK, so it remains to be seen whether similar outcomes would be observed in groups who already know one another, or in an in-person setting. More particularly, given that peer-led school interventions targeting youth mental health are often used in schools, despite the sparse evidence-base, adapting this intervention and testing its efficacy in school settings, either online or offline, would help advance the field. It is, however, worth noting that feedback from both YPAGs who supported this project, and initial evidence from our qualitative results, suggested that having the intervention delivered to groups of strangers made participants more comfortable to talk about mental health. Researchers working on peer-led school interventions should investigate whether and why the school environment might pose barriers to young people feeling safe to ‘open up’ and share emotional difficulties.

## Limitations

Because the existing evidence on any impact of peer-led interventions is limited and inconclusive [22, 23], and this was the first evaluation of this intervention, we did not assess medium to long-term effects, and only compared the intervention to a passive control condition. Further studies are needed to examine the enduring effects of the intervention, and their magnitude in relation to active controls. This is particularly important given that we found larger effects immediately after the intervention than 1 week later, suggesting that effects might fade over time. However, there is evidence that large immediate gains predict longer-term benefits of brief online interventions targeting emotional coping skills in adolescence [44]. Moreover, our sample was diverse but overly represented by females. Future research should investigate ways to make this intervention more appealing to male participants. Finally, engagement with our online intervention may have been aided by restrictions on in-person contact imposed by COVID. To gain a comprehensive understanding of the intervention’s acceptability and adherence, future research should extend its investigation beyond a social distancing context.

## Conclusion

Working together with a peer support charity and a group of adolescents trained in peer support, we co-created and evaluated an online peer-led intervention to support young people’s wellbeing through the challenges of the COVID-19 pandemic. This peer-led intervention led to benefits in young people’s wellbeing, with additional gains to their social connectedness, coping skills, sense of purpose, self-esteem and self-compassion, compared to wait-list controls. These findings suggest that online peer-led interventions can be an important tool to help support young people’s mental health and wellbeing during a health emergency.

### Supplementary Information

**Additional file 1. **Description of data Supplementary tables containing details of YPAG contributions and results.

**Additional file 2. **Description of Coping during COVID programme.

## Data Availability

The dataset and codebook are available on the Open Science Framework (https://osf.io/s7wnj/?view_only=37c8a45fb90d43469b1d43ae8d503828). Qualitative data and research materials are available from the corresponding author upon reasonable request.

## Footnotes

1 Due to a technical problem related to the use of the automated randomisation tool, n slightly differed between conditions.

## References

[1] Dewa LH, Crandell C, Choong E (2021). CCopeY: a mixed-methods coproduced study on the mental health status and coping strategies of young people during COVID-19 UK Lockdown. J Adolesc Heal.

[2] Loades ME, Chatburn E, Higson-Sweeney N (2020). Rapid systematic review: the impact of social isolation and loneliness on the mental health of children and adolescents in the context of COVID-19. J Am Acad Child Adolesc Psychiatry.

[3] Pierce M, Hope H, Ford T (2020). Mental health before and during the COVID-19 pandemic: a longitudinal probability sample survey of the UK population. Lancet Psychiatry.

[4] Smith A (2007). Children as social actors: an introduction. Int J Child Rights.

[5] Larcher V, Dittborn M, Linthicum J (2020). Young people’s views on their role in the COVID-19 pandemic and society’s recovery from it. Arch Dis Child.

[6] Pavarini G, Lyreskog D, Manku K (2020). Promoting capabilities for young people’s agency in the COVID-19 outbreak. Child Adolesc Ment Health.

[7] Racine N, McArthur BA, Cooke JE (2021). Global prevalence of depressive and anxiety symptoms in children and adolescents during COVID-19. JAMA Pediatr.

[8] Houlston C, Smith PK, Jessel J (2009). Investigating the extent and use of peer support initiatives in english schools. Educ Psychol.

[9] Naylor P, Cowie H (1999). The effectiveness of peer support systems in challenging school bullying: the perspectives and experiences of teachers and pupils. J Adolesc.

[10] Visser MJ (2004). Implementing peer support in schools: using a theoretical framework in action research. J Community Appl Soc Psychol.

[11] Naslund JA, Aschbrenner KA, Marsch LA (2016). The future of mental health care: peer-to-peer support and social media. Epidemiol Psychiatr Sci.

[12] Galea S, Merchant RM, Lurie N (2020). The mental health consequences of COVID-19 and physical distancing. JAMA Intern Med.

[13] Torous J, Jän Myrick K, Rauseo-Ricupero N (2020). Digital mental health and COVID-19: using technology today to accelerate the curve on access and quality tomorrow. JMIR Ment Heal.

[14] Suresh R, Alam A, Karkossa Z (2021). Using peer support to strengthen mental health during the COVID-19 pandemic: a review. Front Psychiatry.

[15] Andrews JL, Foulkes L, Blakemore S-J (2020). Peer influence in adolescence: public-health implications for COVID-19. Trends Cogn Sci.

[16] Vijayakumar N, Pfeifer JH (2020). Self-disclosure during adolescence: exploring the means, targets, and types of personal exchanges. Curr Opin Psychol.

[17] Boldero J, Fallon B (1995). Adolescent help-seeking: what do they get help for and from whom?. J Adolesc.

[18] Steiner RJ, Sheremenko G, Lesesne C (2019). Adolescent connectedness and adult health outcomes. Pediatrics.

[19] Yorke C, Reardon T, Mawdsley G, et al. Equals and role-models: A qualitative analysis of how adolescents perceive peer leaders in an online wellbeing program during the COVID-19 pandemic. 2024. Manuscript submitted for publication.

[20] Choukas-Bradley S, Giletta M, Cohen GL (2015). Peer influence, peer status, and prosocial behavior: an experimental investigation of peer socialization of adolescents’ intentions to volunteer. J Youth Adolesc.

[21] Foulkes L, Leung JT, Fuhrmann D (2018). Age differences in the prosocial influence effect. Dev Sci.

[22] King T, Fazel M (2021). Examining the mental health outcomes of school-based peer-led interventions on young people: a scoping review of range and a systematic review of effectiveness. PLoS ONE.

[23] Ali K, Farrer L, Gulliver A (2015). Online peer-to-peer support for young people with mental health problems: a systematic review. JMIR Ment Heal.

[24] Ginossar T, Nelson S (2010). Reducing the health and digital divides: a model for using community-based participatory research approach to e-health interventions in low-income hispanic communities. J Comput Commun.

[25] Petersen C, Adams SA, DeMuro PR (2015). mHealth: don’t forget all the stakeholders in the business case. Med.

[26] Puspakesuma N, Kerasidou A, Pavarini G. Children and young people’s voices in digital mental health: an analysis using epistemic injustice. In: Hendl T, Jansky B, Wild V, et al. editors. mHealth: intersectional ethics for a global society. Oxford: Oxford University Press; Forthcoming.

[27] Hagen P, Collin P, Metcalf A., Nicholas M, Rahilly K, Swainston N. Participatory design of evidence-based online youth mental health promotion, intervention and treatment. 2012. www.youngandwellcrc.org.au/wp-content/uploads/2014/03/Young_and_Well_CRC_IM_PD_Guide.pdf

[28] Fazel M, Hoagwood K (2021). School mental health: integrating young people’s voices to shift the paradigm. Lancet Child Adolesc Heal.

[29] Singh I, Pavarini G, Juma D (2020). Correspondence: multidisciplinary research priorities for the COVID-19 pandemic. Lancet Psychiatry.

[30] Pavarini G, Reardon T, Hollowell A (2023). Online peer support training to promote adolescents emotional support skills mental health and agency during COVID-19 randomised controlled trial and qualitative evaluation. Eur Child Adolesc Psychiatry.

[31] Pavarini G, Lorimer J, Manzini A (2019). Co-producing research with youth: the NeurOx young people’s advisory group model. Heal Expect.

[32] Tennant R, Hiller L, Fishwick R (2007). The warwick-edinburgh mental well-being scale (WEMWBS): development and UK validation. Health Qual Life Outcomes.

[33] Lee RM, Robbins SB (1995). Measuring belongingness: the social connectedness and the social assurance scales. J Couns Psychol.

[34] Ladouceur CD. COVID-19 Adolescent Symptom & Psycho- logical Experience Questionnaire. 2020. https://www.nlm.nih.gov/dr2/CASPE_AdolSelfReport_Qualtrics.pdf

[35] Bronk KC, Riches BR, Mangan SA (2018). Claremont purpose scale: a measure that assesses the three dimensions of purpose among adolescents. Res Hum Dev.

[36] Rosenberg M (1965). Society and the adolescent self-image princeton.

[37] Gilbert P, Catarino F, Duarte C (2017). The development of compassionate engagement and action scales for self and others. J Compassionate Heal Care.

[38] O’Connor C, Joffe H (2020). Intercoder reliability in qualitative research: debates and practical guidelines. Int J Qual Methods.

[39] Currie C, Roberts C, Morgan A (2004). Young people’s health in context.

[40] Orben A, Tomova L, Blakemore S-J (2020). The effects of social deprivation on adolescent development and mental health. Lancet Child Adolesc Heal.

[41] Ellis LA, Campbell AJ, Sethi SOB (2011). Comparative randomized trial of an online cognitive-behavioral therapy program and an online support group for depression and anxiety. J Cyber Ther Rehabil.

[42] Woodruff SI, Conway TL, Edwards CC (2007). Evaluation of an Internet virtual world chat room for adolescent smoking cessation. Addict Behav.

[43] Hughes FRA, Botfield JR, McGeechan K (2021). Sexual and reproductive health train the trainer programs in low- and middle-income countries: a scoping review. J Glob Heal Rep.

[44] Schleider JL, Abel MR, Weisz JR (2019). Do Immediate gains predict long-term symptom change? findings from a randomized trial of a single-session intervention for youth anxiety and depression. Child Psychiatry Hum Dev.

