# Supplementary material for: Online peer-led intervention to improve adolescent wellbeing during the COVID-19 pandemic: a randomised controlled trial
Source: Child Adolesc Psychiatry Ment Health. 2024 Mar 18;18:36. doi: 10.1186/s13034-024-00723-1 (PMC10949785; doi:10.1186/s13034-024-00723-1)
Supplement: Supplementary file 1 — Additional file 1. Description of data Supplementary tables containing details of YPAG contributions and results. [file 13034_2024_723_MOESM1_ESM.docx]

Additional file 1 for

**Online peer-led intervention to improve adolescent wellbeing during the COVID-19 pandemic: A randomised controlled trial**

Gabriela Pavarini*, Tessa Reardon, Geoffrey Mawdsley, Ilina Singh

*Corresponding author: gabriela.pavarini@ethox.ox.ac.uk

**This file includes:**

TableS1

TableS2

**Table S1.** Youth involvement in the trial from NeurOX YPAG and Uplift YPAG

| **Number of meetings** | **Group** | **Date** | **Contribution** |
| --- | --- | --- | --- |
| 1 | NeurOx YPAG | 20/10/2020 | The NeurOx YPAG answered questions surrounding the study including which groups of young people were most affected by the COVID-19 pandemic in the UK, how to reach this group for recruitment, difficulties of running an intervention in November/December, their thoughts on peer support programmes. |
| 1 | Uplift YPAG | 16/10/2020 | The Uplift YPAG were consulted on ‘what do you feel your friends and peers are struggling with the most at the moment’?. Common themes that emerged surrounded isolation, lack of purpose, adapting to new ways of living such as online schooling, concerns about the future, concerns for others, and concerns about young people’s perceptions and identity. |
| 2 | Uplift YPAG | 21/10/2020  30/10/2020 | Codesign sessions run with Uplift YPAG whereby they contributed to the planned recruitment strategies and study methodology. This included designing Instagram adverts, reflections on RCT design, methods to communicate with participants, and methods of data collection. |
| 1 | NeurOx YPAG | 22/10/2020 | A NeurOx YPAG meeting centred around the focus of the intervention, what content should be included, which constructs should be targeted and how they should be measured, logistical suggestions of running a course, how the relationship between group leaders and course attendees should be characterised. |
| 1 | Uplift YPAG | 04/11/2020 | Social media recruitment advertisement with Uplift YPAG via WhatsApp. Feedback included bright colours, simple lexicon, plain font, and Oxford University logo for credibility. |
| 1 | Uplift YPAG | 7/11/2020 | Emotion consultation with 17 of the Uplift YPAG via WhatsApp. Asked what were the main emotions their peers were experiencing during the second November lockdown. Main emotions included anxiety, frustration, powerlessness, loneliness, and meaningless. |
| 2 | NeurOx YPAG and Uplift YPAG | 20/11/2020  04/02/2021 | NeurOx YPAG feedback session on mental health promotion initiatives they have been exposed to, discussion on whether they thought they had space or opportunities to voice their views on matters that affect them, and views on digital innovation. |
| 1 | NeurOx YPAG and Uplift YPAG | 04/02/2021 | NeurOx YPAG and Uplift YPAG together were consulted on how the results of the trial could help inform a design of the course that could be sustainably implemented. Ideas included implementation in schools and sixth forms whereby nominated students could receive formal training and deliver the content in their own schools. National integration was viewed as important as the current programme allowed the youth to foster new connections. Therefore creating a wider network of young people across the country to share resources, ideas, and create new connections was proposed. |
| 1 | NeurOx YPAG | 25/05/2021 | GM and GP to present the results to 2 NeurOx YPAG members to discuss the findings. They felt the results were valid but repeating the intervention in a non-COVID context would also be important as well as having more male representation. They believed a key finding was the success of the online format. |
| 1 | Uplift YPAG | 20/07/2021 | GM and GP presented the results from the trial to the Uplift YPAG and asked (1) whether the results make sense; (2) what about the programme drove the observed improvements; and (3) what else is there to find out. They believed the results made sense and believed the effects were driven by an openness and motivation to attend to learn the content, and they were interested to see whether the results would hold with more male representation and in a face-to-face context. |

**Table S2.**

Linear mixed effect models estimates (and 95% CI) for primary and secondary outcomes

|  | **Wellbeing** | **Social connectedness** | **Perceived coping** | **Self-esteem** | **Sense of purpose (total)** | **Self-compassion (total)** |
| --- | --- | --- | --- | --- | --- | --- |
| Group  Intervention vs wait-list | 4.53 (2.78-6.28), p<0.001 | 7.71 (4.76-10.66), p<0.001 | 0.96 (0.40-1.52),  p=0.001 | 2.11 (1.21-3.01), p<0.001 | 5.36 (3.64-7.07), p<0.001 | 4.56 (1.44-7.67), p=0.005 |
| Timepoint | *p<0.001 | *p<0.001 | *p<0.001 | *p<0.001 | *p<0.001 | *p=0.002 |
| Group by timepoint interaction | *p<0.001 | *p<0.001 | *p=0.001 | *p<0.001 | *p<0.001 | *p=0.004 |
| **Intervention vs wait-list (1-week post-randomisation)** | **8.87 (6.38-11.35), p<0.001** | **16.31 (12.07-20.55), p<0.001** | **1.76 (0.94-2.57), p<0.001** | **4.07 (2.74-5.40), p<0.001** | **9.53 (7.09-11.96), p<0.001** | **7.61 (3.31-11.91), p=0.001** |
| **Intervention vs wait-list (2-weeks post-randomisation)** | **5.17 (2.70-7.63), p<0.001** | **6.53 (2.32-10.75), p=0.003** | **1.21 (0.40-2.02), p=0.004** | **2.28 (0.96-3.61), p=0.001** | **6.73 (4.28-9.19), p<0.001** | **6.27 (1.99-10.55), p=0.004** |
| Gender  Female vs other genders | 2.05 (-0.27-4.37), p=0.083 | 2.25 (-1.68-6.18), p=0.258 | 0.01 (-0.74-0.76), p=0.98 | 0.09 (-1.11-1.29), p=0.88 | 1.40 (-0.90-3.69), p=0.23 | 0.81 (-3.37-4.98), p=0.71 |
| Age  16 years vs 17 or 18 years | -0.96 (-2.82-0.89), p=0.306 | 0.43 (-2.73-3.58), p=0.790 | 0.04 (-0.56-0.64), p=0.90 | 0.32 (-0.65-1.29), p=0.51 | -0.77 (-2.59-1.06), p=0.407 | -1.31 (-4.63-2.02), p=0.44 |
| Corresponding Baseline score | 0.87 (0.75-0.98), p<0.001 | 0.82 (0.74-0.91), p<0.001 | 0.68 (0.56-0.80), p<0.001 | 0.91 (0.82-1.00), p<0.001 | 0.86 (0.77-0.95), p<0.001 | 0.79 (0.66-0.91), p<0.001 |

*where three levels no overall difference provided
