# Supplementary material for: Online peer-led intervention to improve adolescent wellbeing during the COVID-19 pandemic: a randomised controlled trial
Source: Child Adolesc Psychiatry Ment Health. 2024 Mar 18;18:36. doi: 10.1186/s13034-024-00723-1 (PMC10949785; doi:10.1186/s13034-024-00723-1)
Supplement: Supplementary file 2 — Additional file 2. Description of Coping during COVID programme. [file 13034_2024_723_MOESM2_ESM.docx]

Additional file 2 for

**Online peer-led intervention to improve adolescent wellbeing during the COVID-19 pandemic: A randomised controlled trial**

Gabriela Pavarini*, Tessa Reardon, Geoffrey Mawdsley, Ilina Singh

*Corresponding author: gabriela.pavarini@ethox.ox.ac.uk

**This file includes:**

Coping during COVID programme description


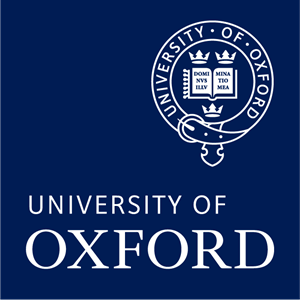

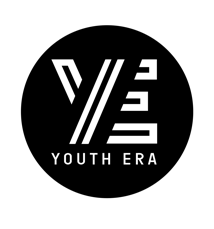


**Coping during COVID Programme**

| **THEME** | **FOCUS** | **ACTIVITIES** |
| --- | --- | --- |
| **The Foundation: Vulnerability & Self-Care** | Vulnerability  Psychological wellbeing  Self-Care  Coping Skills | - **Reveal Yourself:** Participants are presented with scenarios reflecting life experiences. Those identifying with a scenario may join via camera, fostering belonging and peer support through personal narrative sharing. - **The Zone:** Strategies for emotional state management are taught, and participants practice skills during facilitator-prompted scenarios to adjust energy levels. - **Wellness Wheel:** Participants complete a visual wellness or self-care assessment across multiple dimensions. Participants reflect on their self-assessments and discuss areas for enhancement. - **Coping Skills Worksheet:** Participants generate coping strategies for a variety of scenarios and discuss coping mechanisms for navigating life challenges within their groups. |
| **Living a Purpose-Driven Life** | Mission & Purpose  Values | - **Your Why:** Participants reflect on why they joined the program. This discussion aims to build community, increase engagement, and enhance participants' learning experience. - **Mission control:** Participants fill out a worksheet to reflect on their purpose, principles, and goals. This activity encourages introspection and helps connect to personal objectives and values. |
| **Unlocking Your True Potential** | Leadership  Identity & Values  Visioning | - **Future Self:** Participants do a self-visualization aimed at enhancing motivation and personal growth. This activity aids in clarifying goals, increasing motivation, and identifying the steps needed to bridge the gap between current and future states. - **Personality Profile:** Participants complete a worksheet to reflect on their roles in team dynamics and leadership styles. Activity helps improve interpersonal skills and team collaboration. |
| **Be Who You Needed** | Strengths & Assets  Empowerment | - **Reframe:** Participants explore the shift from deficit-based to asset-based thinking. They are presented with scenarios to practice identifying strengths and applying asset-based reframing. - **Empowering Beliefs:** Involves a visualization process aimed at addressing negative belief systems and their impact. Participants are guided to embrace empowering beliefs to enhance mental well-being. |
| **Uplifting Your Peers and Community** | Validation & Affirmation  Lived Experience  Active Listening | - **Live View:** Participants take turns being the focus in small groups, receiving and only responding with "thank you" to open-ended questions or affirmations from peers. Afterward, they share their experience. Activity aims to boost confidence and practicing strength recognition. - **Lived Experience Iceberg:** Participants complete a worksheet on personal experiences, noting what they're comfortable sharing with peers. Activity promotes self-awareness about sharing boundaries and enhances understanding of personal and shared identities. - **Listening Levels:** Participants watch a video to identify different listening levels used, then discuss personal experiences with these listening levels and their impact. |
